# Modulation of Progesterone Receptor Isoform Expression in Pregnant Human Myometrium

**DOI:** 10.1155/2017/4589214

**Published:** 2017-05-02

**Authors:** Marina Ilicic, Tamas Zakar, Jonathan W. Paul

**Affiliations:** ^1^School of Medicine and Public Health, Faculty of Health and Medicine, University of Newcastle, Callaghan, NSW 2308, Australia; ^2^Priority Research Centre for Reproductive Science, University of Newcastle, Callaghan, NSW 2308, Australia; ^3^Hunter Medical Research Institute, 1 Kookaburra Circuit, New Lambton Heights, NSW 2305, Australia; ^4^John Hunter Hospital, New Lambton Heights, NSW 2305, Australia

## Abstract

*Background.* Regulation of myometrial progesterone receptor (PR) expression is an unresolved issue central to understanding the mechanism of functional progesterone withdrawal and initiation of labor in women.* Objectives.* To determine whether pregnant human myometrium undergoes culture-induced changes in* PR* isoform expression ex situ and, further, to determine if conditions approaching the in vivo environment stabilise* PR* isoform expression in culture.* Methods.* Term nonlaboring human myometrial tissues were cultured under specific conditions: serum supplementation, steroids, stretch, cAMP, PMA, PGF_2*α*_, NF-*κ*B inhibitors, or TSA. Following 48 h culture,* PR-T*,* PR-A*, and* PR-B* mRNA levels were determined using qRT-PCR.* PR-A*/*PR-B* ratios were calculated.* Results. PR-T* and* PR-A* expression and the* PR-A*/*PR-B* ratio significantly increased in culture. Steroids prevented the culture-induced increase in* PR-T* and* PR-A* expression. Stretch blocked the effects of steroids on* PR-T* and* PR-A* expression. PMA further increased the* PR-A*/*PR-B* ratio, while TSA blocked culture-induced increases of* PR-A* expression and the* PR-A*/*PR-B* ratio.* Conclusion.* Human myometrial tissue in culture undergoes changes in* PR* gene expression consistent with transition toward a laboring phenotype. TSA maintained the nonlaboring PR isoform expression pattern. This suggests that preserving histone and/or nonhistone protein acetylation is critical for maintaining the progesterone dependent quiescent phenotype of human myometrium in culture.

## 1. Introduction

Preterm birth is a major societal and economic problem that affects approximately 9.6% of pregnancies worldwide and accounts for 80–90% of neonatal morbidity and death [1–4]. The prevention of preterm birth continues to be an important health priority. There is a substantial body of evidence highlighting the importance of progesterone in maintaining the pregnant state by promoting myometrial quiescence and relaxation [5–7]. The withdrawal of progesterone action signals the end of pregnancy and in most mammalian species happens by a rapid fall in circulating levels of progesterone [8–12]. In humans and higher primates, however, maternal, fetal, and amniotic concentrations of progesterone remain elevated during parturition and delivery, suggesting that systemic progesterone withdrawal does not occur at the initiation of labor [13–15]. Nonetheless, the administration of a synthetic progesterone antagonist, RU486, to humans at any stage of pregnancy promotes cervical ripening and parturition [5, 6, 16–19]. As such a “functional” withdrawal of progesterone action has been proposed to explain the loss of propregnancy progesterone actions despite circulating levels of progesterone remaining elevated. The exact mechanism of functional progesterone withdrawal is unclear and in recent years it has been the focus of intense research. One proposed mechanism is that functional progesterone withdrawal occurs through a decrease in myometrial responsiveness caused by a change in progesterone receptor (PR) isoform expression. Two major isoforms, PR-A and PR-B, exist in humans. PR-B is the principal transcriptional mediator of progesterone action and maintains uterine quiescence, while PR-A represses the transcriptional activity of PR-B and therefore decreases progesterone responsiveness [3, 5, 6, 19]. Recent work has also shown that PR-A ligand-independently stimulates the expression of the key labor promoting gene* Cx43* [20]. Thus, genomic progesterone responsiveness is believed to be regulated by the opposing actions of PR-A and PR-B and is inversely associated with the PR-A/PR-B ratio [3, 5, 6, 19]. Indeed, several studies, including our own, have shown that myometrial expression of* PR-A* has significantly increased late in human pregnancy and with the onset of labor [21–24].

Elucidating the mechanism of functional progesterone withdrawal is therefore important for understanding the mechanisms regulating the balance between uterine quiescence and contractions. Outside of clinical trials, researchers are primarily limited to observational studies on human pregnancy. Interventional studies rely on animal models of pregnancy as well as on in vitro experiments using human myometrial smooth muscle cell lines and tissues. Human cell cultures are a valuable in vitro tool used to gain insight into numerous physiological and pathological processes; however, concerns have been raised about the lifespan of cultured primary cells [25] as well as their ability to remain to be representative of the tissue of origin [26–29]. The use of ex vivo myometrial tissue may represent the in vivo phenotype more closely and can involve utilizing smooth muscle biopsy samples as small pieces or dissecting the tissue into strips. Tissue strips are primarily utilized to examine myometrial contractility [30–33] such as the dynamic phosphorylation events that occur in phase with contractions [34, 35].

Although the use of ex vivo tissues pieces and strips has greatly facilitated studies into gene expression and regulation, both approaches rely on the assumption that the tissue phenotype remains stable across the course of the study. For instance, it is assumed that nonlaboring myometrium retains a nonlaboring phenotype ex vivo providing an experimental system to induce labor-associated changes. Myometrial strips from nonlaboring pregnant women, however, spontaneously develop contractions ex vivo over the course of just 1-2 h, suggesting a rapid transition away from the nonlaboring in vivo phenotype [30, 33, 35]. Furthermore, tissue incubation studies are routinely performed for 48 h or more; therefore the transition away from the original phenotype may be even more pronounced after 48 h culture in vitro.

The aim of this study was to determine if nonlaboring myometrial tissue pieces and strips undergo culture-induced changes in PR expression that are consistent with transition to a PR isoform expression pattern similar to labor. We further aimed to identify culture conditions that could be implemented to block or minimize such transition in vitro, presenting researchers with a stable platform on which to conduct experimental studies.

Here we report that nonlaboring human myometrium undergoes culture-induced changes in* PR* isoform expression in vitro comparable with the changes attributed to functional progesterone withdrawal at labor. We further report that supplementing media with the histone deacetylase inhibitor (HDACi), trichostatin A (TSA), prevents the culture-induced functional progesterone withdrawal phenomenon by maintaining a low* PR-A/PR-B* ratio, consistent with maintenance of a nonlaboring phenotype.

## 2. Materials and Methods

### 2.1. Consumables and Reagents

Superscript III First-Strand Synthesis System, Ultrapure Glycogen, UltraPure Agarose, and Trackit 100 BP DNA ladder were purchased from Invitrogen (Carlsbad, USA). TRizol Reagent and Turbo DNA-free 50 reactions were from Ambion (Thermo Fisher). Alien QRT-PCR Inhibitor Alert 400 Reactions were purchased from Integrated Sciences Pty (Sydney, Australia). (R)-MG132, BAY-11-7085, Phorbol Myristate Acetate (PMA), and Prostaglandin F_2*α*_ (PGF_2*α*_) were obtained from Cayman Chemical Company (Michigan, USA). 8-Bromoadenosine 3′,5′-cyclic monophosphate (8-Br-cAMP), PCR primers, progesterone, and estradiol were purchased from Sigma (St Louis, USA). 2 mL 2.8 mm ceramic bead kits (CK28-R) for the Precellys homogenizer (Bertin Instruments, France) were purchased from Thermo Fischer Scientific (Melbourne, Australia). L-Glutamine, Sodium Pyruvate, Gentamicin, HEPES, Dulbecco's Modified Eagle Medium (DMEM), and Charcoal Stripped Fetal Bovine Serum were obtained from Gibco (Carlsbad, USA). SYBR Green 2x Master mix was from Applied Biosystems (Carlsbad, USA). TSA was supplied by Bio-Scientific Pty. Ltd. (Sydney, Australia).

### 2.2. Myometrial Tissue Acquisition

These studies were approved by the Hunter and New England Area Human Research Ethics Committee and the University of Newcastle Human Ethics Committee (02/06/12/3.13). Human myometrial samples were obtained from the lower uterine segment during elective Caesarean section (CS) of singleton term pregnancies (38.2–39.6 weeks' gestation). Patient body mass index (BMI) range was 18.3–38.0, and none of the patients were in-labor. The indications for elective CS were previous CS, placenta praevia, fetal distress, or breach presentation. Women were excluded if they were given steroids. Following delivery of the placenta, 5 units of syntocinon were administrated directly into an intravenous line as part of standard care for the prevention of postpartum hemorrhage. Samples were therefore exposed to oxytocin for a brief period of time (3 min). All samples were placed on ice in serum-free medium containing DMEM with high glucose, 2 mM L-Glutamine, 1 mM Sodium Pyruvate, 40 *μ*g/mL Gentamicin, and 10 mM HEPES for the transfer to the laboratory.

### 2.3. Myometrial Tissue (Explant) Culture

Approximately 100 mg tissue from each sample was immediately snap-frozen in liquid nitrogen for subsequent analysis. The remaining myometrium was dissected into approximately 2 × 2 × 2 mm pieces and washed in serum-free media. Samples were then incubated in serum-free or 5% (v/v) charcoal stripped serum- (CSS-) supplemented media in a 37°C, 95% air/5% CO_2_ humidified incubator for 48 h. The 5% CSS-supplemented media contained DMEM with high glucose, 5% CSS, 2 mM L-Glutamine, 1 mM Sodium Pyruvate, 40 *μ*g/mL Gentamicin, and 10 mM HEPES. To determine the effects of steroids, myometrial samples were incubated with physiological concentrations of progesterone (P4; 500 nM) and/or estradiol (E2; 400 nM) [14] in a 37°C, 95% air/5% CO_2_ humidified incubator for 48 hours. To determine the effect of stretch on human myometrium, myometrial tissue strips (2 × 2 × 10 mm) were cultured in 5% CSS-containing media for 48 h in a 37°C, 95% air/5% CO_2_ humidified incubator while being subjected to 0, 1, or 3 g of constant stretch. Constant stretch was applied by using nylon thread to attach stainless steel weights to the ends of strips and then suspending the strips in 30 mL of culture media in 50 mL tubes (strips subjected to 0 g only were tied at one end). To determine the effect of stretch and steroids on human myometrium, myometrial strips were cultured in 5% CSS-containing media with 500 nM P4 and 400 nM E2 for 48 h in a 37°C, 95% air/5% CO_2_ humidified incubator while being subjected to 0, 1, or 3 g of stretch. To determine the effects of the signalling pathways involved in myometrial relaxation and contraction, myometrial tissues were incubated for 48 h a 37°C, 95% air/5% CO_2_ in 5% CSS-containing media supplemented with the cAMP analogue 8-Br-cAMP (250 *μ*M), PMA (0.1, 1.0 *μ*M), PGF_2*α*_ (1, 10, 100, and 100 nM), or the nuclear factor-*κ*B (NF-*κ*B) inhibitors MG-132 (2.0, 5.0, and 10.0 *μ*M) and BAY-11-7085 (2.0, 5.0, and 10.0 *μ*M) individually or in combination (10.0 *μ*M MG-132 + 10.0 *μ*M BAY-11-7085). Furthermore, myometrial tissues were incubated for 48 h a 37°C, 95% air/5% CO_2_ in 5% CSS-containing media supplemented with TSA (0.5, 1.0, 2.5, or 5.0 *μ*M). Vehicle was DMSO (0.1%). Following each incubation, the media were decanted and tissue pieces or strips were snap-frozen using liquid nitrogen and stored at −80°C for subsequent analyses.

### 2.4. RNA Extraction, Reverse Transcription, and Real-Time Quantitative PCR

RNA was extracted from 100 mg of tissue using TRizol Reagent (Thermo Fisher) according to the manufacture's protocol. Homogenization of tissue in TRizol Reagent was performed using a Precellys 24 homogenizer (Bertin Instruments, France). Following extraction, RNA samples were purified using the TURBO DNA-*free* kit (Thermo Fisher). An ND-1000 spectrophotometer was used to measure RNA concentration (absorbance at 260 nm (A_260_) and 280 nm (A_280_)) and purity. RNA integrity was checked by agarose gel electrophoresis. Each RNA sample (0.5 *μ*g of total RNA) was spiked with 0.5 × 10^7^ copies of Alien RNA and reverse-transcribed using the SuperScript III First-Strand Synthesis System with random hexamer primers. Quantitative RT-PCR was performed using an ABI 7500 Sequence Detector. No-reverse transcription (no-RT) negative controls were prepared for each sample to ensure there was no DNA contamination. The final volume of each PCR reaction was 20 *μ*L containing 10 *μ*L of 2x SYBR Green PCR Master Mix (Thermo Fisher), master mix cDNA template (corresponding to 10 ng of reverse transcribed RNA), target cDNA-specific forward and reverse primers, and MilliQ water. For the reference gene (Alien primer), the final volume was also 20 *μ*L with 1.0 *μ*L of 2.5 *μ*M of Alien Primer Mix, 10 *μ*L of 2x SYBR Green PCR, and the same amount of cDNA as the target genes and MilliQ water. No-template control samples (NTCs) were included in each PCR plate to detect any contamination and primer-dimers. PCR primers were designed using Primer Express and are shown in the Table 1.

### 2.5. Data and Statistical Analysis

All mRNA abundance data were expressed relative to the Alien reference RNA. The relative mRNA abundance was calculated using the delta C_t_ (ΔC_t_) method [36]. The relative mRNA abundance of* PR-A* was calculated by subtracting the relative mRNA abundance of* PR-B* from that of* PR-T*. All mRNA relative abundance values were checked for normal distribution using Shapiro-Wilk normality test and if data was not normally distributed, then it was logarithmically transformed to approach normal distribution. Statistical analyses were conducted with GraphPad Prism software (San Diego, CA, USA). Graphical data are presented as mean ± SEM. For comparison between two groups, Student's *t*-test was used. For multiple comparisons, a one-way analysis of variance (ANOVA) followed by post hoc test of Dunnett multiple comparisons was used. *p* values ≤ 0.05 were considered statistically significant.

## 3. Results

### 3.1. Culture-Induced Changes in Myometrial PR Isoform Expression over Time

Myometrial tissues were incubated for 0, 1, 2, 6, 24, or 48 h in serum-free media to determine changes in* PR* isoform expression that occurred upon being removed from the in vivo environment and cultured in vitro.* PR-T*,* PR-B,* and* PR-A* mRNA abundance were determined across the timeline and the* PR-A/PR-B* ratio calculated.

**Figure 1:**
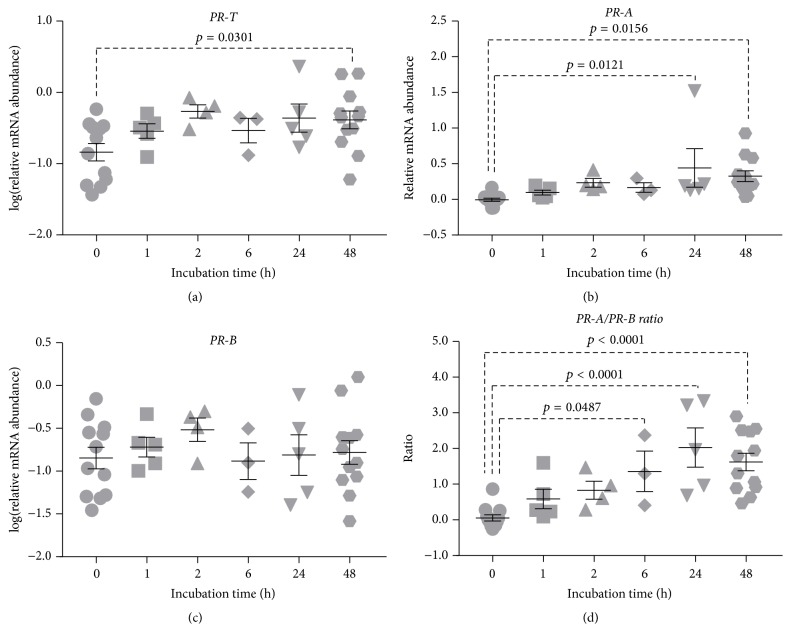
Culture-induced change in myometrial PR isoform expression over time: relative mRNA abundance of* PR-T, PR-A, *and* PR-B *was measured in term nonlaboring myometrial tissue samples at different time points (0, 1, 2, 6, 24, and 48 h), and expressed relative to Alien reference. In addition,* PR-A/PR-B* expression ratio was calculated. (a)* PR-T* mRNA abundance. (b)* PR-A* mRNA abundance. (c)* PR-B* mRNA abundance. (d)* PR-A/PR-B* expression ratio.* Data was checked for normality (Shapiro-Wilk normality test) and if necessary was logarithmically transformed to approach normal distribution (Shapiro-Wilk normality test). Data was analysed using 1-way ANOVA with multiple comparisons (Dunnett)*.* Data are mean ± SEM*.

*PR-T* mRNA abundance was significantly increased after 48 h culture (*p* = 0.0301) (Figure 1(a)) and was attributable to increased* PR-A* mRNA abundance, which was significantly increased beyond 24 h culture (*p* = 0.0121) (Figure 1(b)).* PR-B* mRNA abundance remained relatively constant (Figure 1(c)). The* PR-A*/*PR-B* ratio was significantly increased after 6 h in vitro culture (*p* = 0.0487) and highly significant beyond 24 h culture (*p* < 0.0001) (Figure 1(d)).

### 3.2. Controlling Changes in PR Isoform Expression Using Serum

**Figure 2:**
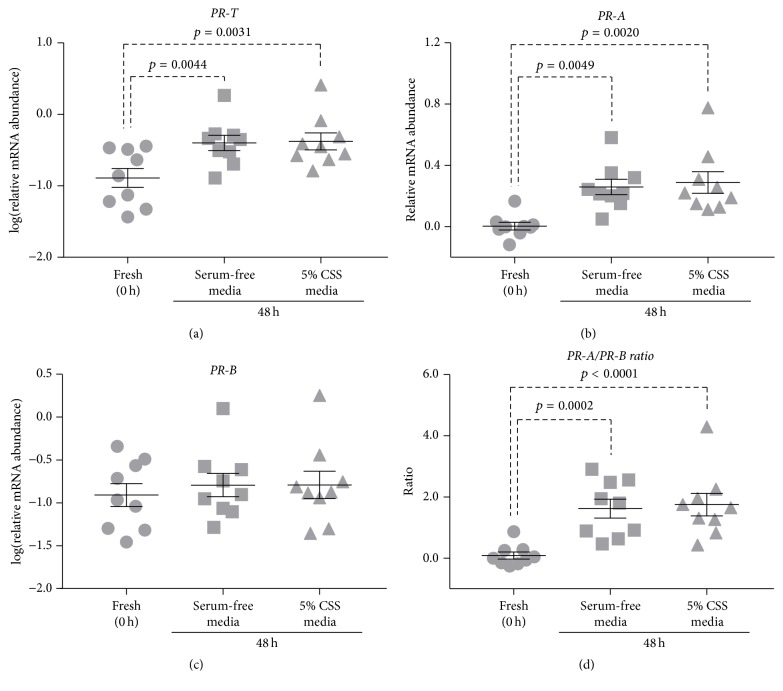
Effect of serum on culture-induced changes in PR isoform expression in vitro: relative mRNA abundance of* PR-T, PR-A,* and* PR-B* was measured in term nonlaboring myometrial tissue samples following 48 h incubation in serum-free media or media supplemented with 5% CSS (*n* = 9) and expressed relative to Alien reference. In addition,* PR-A/PR-B* expression ratio was calculated. (a)* PR-T* mRNA abundance. (b)* PR-A *mRNA abundance. (c)* PR-B* mRNA abundance. (d)* PR-A/PR-B *expression ratio.* Data was checked for normality (Shapiro-Wilk normality test) and if necessary was logarithmically transformed to approach normal distribution (Shapiro-Wilk normality test). Data was analysed using 1-way ANOVA with multiple comparisons (Dunnett)*.* Data are mean ± SEM*.

CSS is often used in myometrial culture media [37–39]. Having observed a culture-induced increase in* PR-A* expression (and thus* PR-T *expression*), *we examined whether supplementing media with 5% CSS affected culture-induced changes in* PR* expression. CSS supplementation had no effect on culture-induced changes in* PR* expression. After 48 h culture, there was no significant difference in* PR-T, PR-A, *or* PR-B *mRNA abundance. Furthermore, there was no significant difference in* PR-A/PR-B* expression ratio between myometrial tissues cultured in serum-free media versus 5% CSS-supplemented media (Figure 2).

### 3.3. Controlling Changes in PR Isoform Expression Using Steroids

Relative abundance of* PR-T, PR-A, *and* PR-B* mRNA was measured in myometrial tissues incubated for 48 h in the presence of 500 nM P4 or 500 nM P4 + 400 nM E2, which are hormone concentrations in term maternal plasma [14].

**Figure 3:**
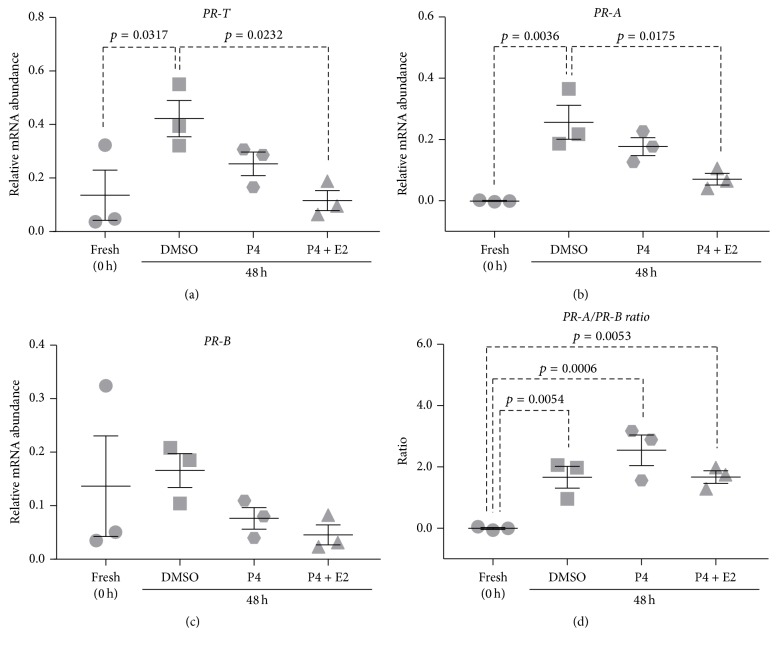
Effect of steroids on culture-induced changes in PR isoform expression in vitro: relative mRNA abundance of* PR-T, PR-A, *and* PR-B* was measured in term nonlaboring myometrial tissue samples following 48 h incubation in the presence of 500 nM progesterone (P4) or 500 nM P4 + 400 nM estradiol (E2) (*n* = 3) and expressed relative to Alien reference. In addition,* PR-A/PR-B* expression ratio was calculated. (a)* PR-T* mRNA abundance. (b)* PR-A* mRNA abundance. (c)* PR-B* mRNA abundance. (d)* PR-A/PR-B* expression ratio.* Data was checked for normality (Shapiro-Wilk normality test) and then analysed using 1-way ANOVA with multiple comparisons (Dunnett). Data are mean ± SEM*.

*PR-T* mRNA abundance significantly increased in DMSO-treated (control) tissues following 48 h incubation (*p* = 0.0317) (Figure 3(a)). Upon supplementing media with 500 nM P4,* PR-T* mRNA abundance was reduced after 48 h culture relative to the control; however, the effect did not reach statistical significance (*p* = 0.2457) (Figure 3(a)). Supplementing media with the combination of 500 nM P4 + 400 nM E2 for 48 h prevented the increase in* PR-T* mRNA abundance to the extent that there was a significant difference relative to 48 h DMSO-treated control tissues (*p* = 0.0232) (Figure 3(a)).

Similarly,* PR-A* mRNA abundance significantly increased in DMSO-treated (control) tissues following 48 h incubation (*p* = 0.0036) and supplementing media with 500 nM P4 reduced* PR-A* mRNA abundance after 48 h relative to the control; however, the difference was not statistically significant (*p* = 0.3234) relative to 48 h DMSO-treated control tissues (Figure 3(b)). Supplementing media with the combination of 500 nM P4 + 400 nM E2 prevented the increase in* PR-A* mRNA abundance to the extent that there was a significant difference relative to 48 h DMSO-treated control tissues (*p* = 0.0175) (Figure 3(b)).

*PR-B* mRNA abundance remained unchanged over 48 h of culture and supplementing media with 500 nM P4 alone, or 500 nM P4 + 400 nM E2, had no significant effect on* PR-B* mRNA abundance (Figure 3(c)).

The* PR-A/PR-B* expression ratio significantly increased in DMSO-treated (control) tissues following 48 h incubation (*p* = 0.0054) (Figure 3(d)). P4-supplementation had no effect on the* PR-A/PR-B* expression ratio relative to DMSO-treated control tissues and remained significantly elevated compared to fresh tissues (*p* = 0.0006) (Figure 3(d)). Similarly, the combination of 500 nM P4 + 400 nM E2 had no significant effect on the* PR-A/PR-B *expression ratio after 48 h relative to DMSO-treated control tissues (*p* > 0.9999), and the* PR-A/PR-B* expression ratio remained significantly elevated relative to the fresh tissues (*p* = 0.0053) (Figure 3(d)).

### 3.4. Controlling Changes in PR Isoform Expression Using Stretch

Myometrial tissue strips were subjected to 0, 1, or 3 g of stretch for 48 h to determine whether applying stretch to the muscle influenced culture-induced changes in* PR *isoform expression. The effect of stretch was investigated in the absence and presence of steroids (500 nM P4 + 400 nM E2).

**Figure 4:**
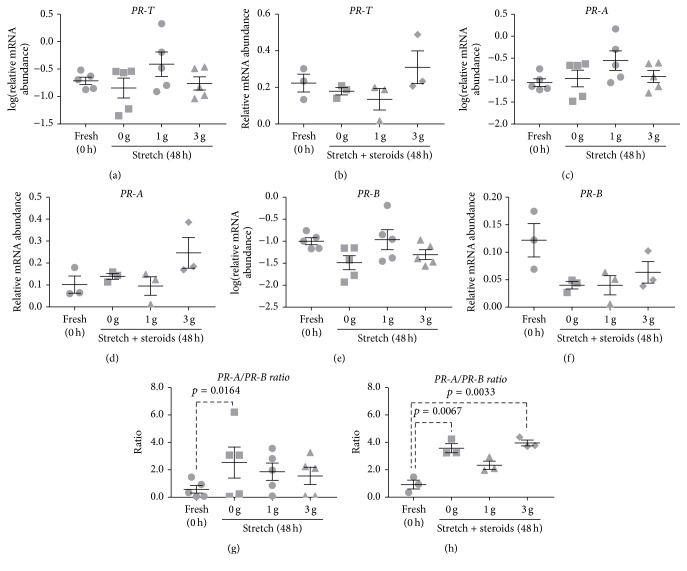
Effect of stretch in the absence or presence of steroids on culture-induced changes in PR isoform expression in vitro: Relative mRNA abundance of* PR-T*,* PR-A,* and* PR-B* was measured in term nonlaboring myometrial strips while applying 0, 1, and 3 g of stretch for 48 h (*n* = 5), as well as in presence of steroids (500 nM P4 + 400 nM E2) while applying 0, 1, and 3 g of stretch for 48 h (*n* = 3), and expressed relative to Alien reference. In addition,* PR-A/PR-B* expression ratio was calculated. (a) Effect of stretch on* PR-T* mRNA abundance. (b) Effect of stretch and steroids on* PR-T* mRNA abundance. (c) Effect of stretch on* PR-A* mRNA abundance. (d) Effect of stretch and steroids on* PR-A* mRNA abundance. (e) Effect of stretch on* PR-B* mRNA abundance. (f) Effect of stretch and steroids on* PR-B* mRNA abundance. (g) Effect of stretch on* PR-A/PR-B* expression ratio. (h) Effect of stretch and steroids on* PR-A/PR-B* expression ratio.* Data was checked for normality (Shapiro-Wilk normality test) and if necessary was logarithmically transformed to approach normal distribution (Shapiro-Wilk normality test). Data was analysed using 1-way ANOVA with multiple comparisons (Dunnett). Data are mean ± SEM*.

Stretch (1 or 3 g) applied to myometrial strips for 48 h had no effect on* PR-T *expression (Figure 4(a)) relative to nonstretched (0 g) control strips. Interestingly, stretch in the presence of 500 nM P4 + 400 nM E2 for 48 h also had no significant effect on* PR-T* mRNA levels (Figure 4(b)), indicating that P4 + E2 was no longer effective in decreasing* PR-T* mRNA abundance compared to fresh tissues.

Similarly, stretch applied to myometrial strips for 48 h had no effect on* PR-A* mRNA expression (Figure 4(c)). Stretch applied in the presence of 500 nM P4 + 400 nM E2 likewise had no effect (Figure 4(d)), indicating that P4 + E2 was no longer effective in preventing culture-induced increase in* PR-A *mRNA abundance compared to fresh tissues.

As seen in Figure 4(e), 0–3 g stretch had no effect on* PR-B* expression in the tissue strips. Stretch in the presence of 500 nM P4 + 400 nM E2 for 48 h also had no significant effect on* PR-B* mRNA levels (Figure 4(f)).

The* PR-A/PR-B* expression ratio was calculated and there was significant increase following 48 h incubation in nonstretched (0 g) control strips when compared to fresh tissues (*p* = 0.0164) (Figure 4(g)). Applying stretch (1 or 3 g) to myometrial strips for 48 h had no effect on expression ratio when compared to nonstretched strips (Figure 4(g)). Further, there was significant increase in* PR-A/PR-B* expression ratio following 48 h incubation in nonstretched (0 g) control strips in the presence of 500 nM P4 + 400 nM E2 when compared to fresh tissues (*p* = 0.0067) (Figure 4(h)). Stretch (1 or 3 g) applied in the presence of 500 nM P4 + 400 nM E2 had no effect on expression ratio after 48 h culture when compared to control strips (Figure 4(h)).

### 3.5. Controlling Changes in PR Isoform Expression Using Cyclic-AMP and PMA

Relative abundance of* PR-T, PR-A, *and* PR-B* mRNA was measured in myometrial tissue incubated for 48 h in the presence of 8-Br-cAMP (250 *μ*M), PMA (0.1 and 1.0 *μ*M), or vehicle (DMSO).

**Figure 5:**
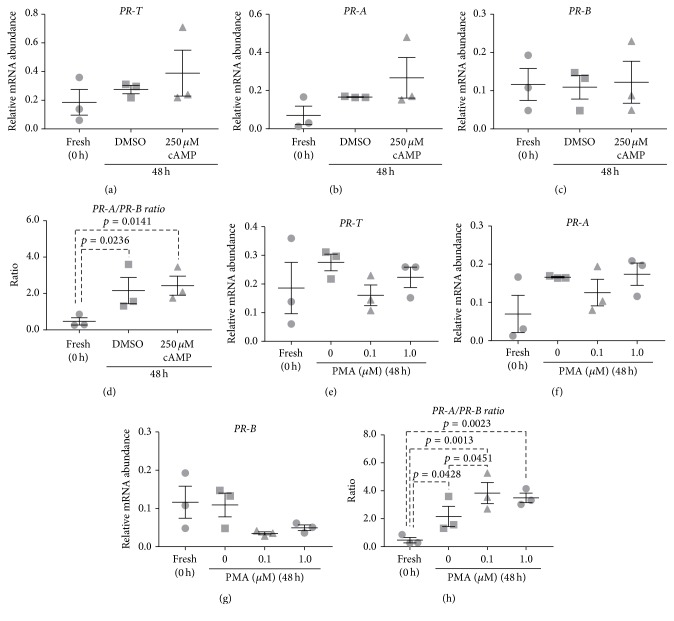
Effect of cAMP and PMA on culture-induced changes in PR isoform expression in vitro: Relative mRNA abundance of* PR-T*,* PR-A, *and* PR-B* was measured in term nonlaboring myometrial tissue samples (*n* = 3) following 48 h incubation in the presence of 8-Br-cAMP (250 *μ*M) or PMA (0.1 and 1.0 *μ*M) and expressed relative to Alien reference. In addition,* PR-A/PR-B* expression ratio was calculated. (a) Effect of 8-Br-cAMP on* PR-T* mRNA abundance. (b) Effect of 8-Br-cAMP on* PR-A* mRNA abundance. (c) Effect of 8-Br-cAMP on* PR-B* mRNA abundance. (d) Effect of 8-Br-cAMP on* PR-A/PR-B* expression ratio. (e) Effect of PMA on* PR-T* mRNA abundance. (f) Effect of PMA on* PR-A* mRNA abundance. (g) Effect of PMA on* PR-B* mRNA abundance. (h) Effect of PMA on* PR-A/PR-B* expression ratio.* Data was checked for normality (Shapiro-Wilk normality test) and then analysed using 1-way ANOVA with multiple comparisons (Dunnett). Data are mean ± SEM*.

Supplementing culture media with 250 *μ*M 8-Br-cAMP had no effect on mRNA abundance for* PR-T*,* PR-A,* or* PR-B *(Figures 5(a)–5(c)). The* PR-A/PR-B* expression ratio in DMSO-treated tissues was significantly elevated following 48 h incubation when compared to fresh tissues (*p* = 0.0236) (Figure 5(d)). Supplementing culture media with 250 *μ*M 8-Br-cAMP did not prevent the increase in the* PR-A/PR-B* expression ratio (*p* = 0.0141) (Figure 5(d)).

Supplementing media with 0.1 or 1.0 *μ*M PMA, a protein kinase C (PKC) activator, had no significant effect on mRNA abundance for* PR-T *or* PR-A, *relative to 48 h DMSO-treated control tissues (Figures 5(e) and 5(f)).* PR-B* mRNA abundance was reduced by both PMA treatments relative to DMSO-treated control tissues; however, the decreases did not reach statistical significance (*p* = 0.3659 and *p* = 0.5259, resp.) (Figure 5(g)). The* PR-A/PR-B* expression ratio was significantly elevated in DMSO-treated control tissues at 48 h, relative to fresh tissues (*p* = 0.0428) (Figure 5(h)). Supplementing media with 0.1 *μ*M PMA significantly increased the* PR-A/PR-B* expression ratio beyond levels detected in the 48 h DMSO-treated control tissues (*p* = 0.0451) (Figure 5(h)) and was attributable to the, albeit nonsignificant, decline in* PR-B* mRNA abundance. The* PR-A/PR-B* expression ratio was significantly elevated after 48 h in 1.0 *μ*M PMA-treated tissues compared to fresh tissues (*p* = 0.0023) (Figure 5(h)).

### 3.6. Controlling Changes in PR Isoform Expression Using PGF_2*α*_

Relative abundance of* PR-T, PR-A, *and* PR-B* mRNA was measured in myometrial tissue incubated for 48 h in the presence of PGF_2*α*_ (1, 10, 100, or 1000 nM) or vehicle (DMSO).

**Figure 6:**
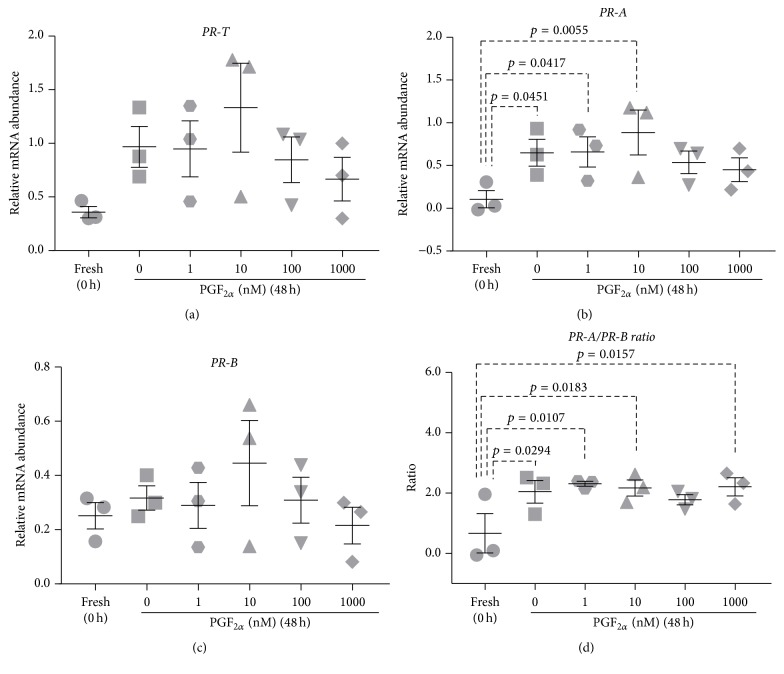
Effect of PGF_2*α*_ on culture-induced changes in PR isoform expression in vitro: relative mRNA abundance of* PR-T, PR-A, *and* PR-B* was measured in term nonlaboring myometrial tissue samples (*n* = 3) following 48 h incubation in the presence of PGF_2*α*_ (1, 10, 100, and 1000 nM) and expressed relative to Alien reference. In addition,* PR-A/PR-B* expression ratio was calculated. (a)* PR-T* mRNA abundance. (b)* PR-A* mRNA abundance. (c)* PR-B* mRNA abundance. (d)* PR-A/PR-B* expression ratio.* Data was checked for normality (Shapiro-Wilk normality test) and then analysed using 1-way ANOVA with multiple comparisons (Dunnett). Data are mean ± SEM*.

Following 48 h incubation* PR-T* mRNA abundance in DMSO-treated control tissues was elevated relative to fresh tissues but did not reach statistical significance (Figure 6(a)). PGF_2*α*_ treatments had no effect on* PR-T* mRNA abundance relative to 48 h DMSO-treated control tissues (Figure 6(a)).

*PR-A* mRNA abundance was significantly increased in DMSO-treated control tissues relative to fresh tissues (*p* = 0.0451) (Figure 6(b)). PGF_2*α*_ treatments had no effect on PR-A mRNA abundance relative to 48 h DMSO-treated control tissues (Figure 6(b)).

*PR-B *mRNA abundance remained unchanged following 48 h incubation and was not affected by PGF_2*α*_ treatments (Figure 6(c)).

In 48 h DMSO-treated tissue the* PR-A/PR-B* expression ratio was significantly elevated relative to fresh tissues (*p* = 0.0294) (Figure 6(d)). PGF_2*α*_ supplementation (1, 10, 100, or 1000 nM) had no effect on the* PR-A/PR-B* expression ratio relative to DMSO-treated control tissues (Figure 6(d)).

### 3.7. Controlling Changes in PR Isoform Expression Using NF-*κ*B Inhibitors

The NF-*κ*B inhibitors, MG-132 and BAY-11-7085, were employed to test whether NF-*κ*B pathway activation was involved in the* PR* isoform expression changes induced by in vitro culture.

**Figure 7:**
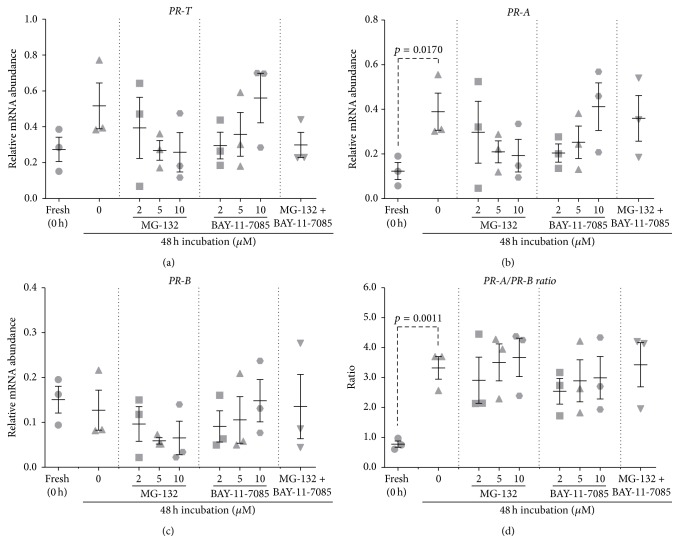
Effect of NF-*κ*B inhibitors, MG-132 and BAY-11-7085, on culture-induced changes in PR isoform expression in vitro: relative mRNA abundance of* PR-T*,* PR-A,* and* PR-B* was measured in term nonlaboring myometrial samples (*n* = 3) following 48 h incubation in the presence of different NF-*κ*B inhibitors and expressed relative to Alien reference RNA. In addition,* PR-A/PR-B* expression ratio was calculated. (a)* PR-T* mRNA abundance. (b)* PR-A* mRNA abundance. (c)* PR-B* mRNA abundance. (d)* PR-A/PR-B* expression ratio.* Data was checked for normality (Shapiro-Wilk normality test) and then analysed using 1-way ANOVA with multiple comparisons (Dunnett). Data are mean ± SEM*.

Following 48 h incubation, there was no significant difference in* PR-T* mRNA abundance between vehicle-treated tissues and fresh tissues (Figure 7(a)). Incubating myometrial samples with 2.0, 5.0, or 10.0 *μ*M MG-132 or BAY-11-7085 individually or in combination (10 *μ*M each) had no effect on* PR-T *mRNA abundance relative to vehicle-treated tissues (Figure 7(a)).

*PR-A* mRNA abundance in vehicle-treated tissues significantly increased compared to fresh tissues following 48 h incubation (*p* = 0.0170) (Figure 7(b)). Supplementing culture media with 2.0, 5.0, or 10.0 *μ*M MG-132 or BAY-11-7085 individually or in combination (10 *μ*M each) had no significant effect on* PR-A *mRNA abundance relative to 48 h vehicle-treated tissues (Figure 7(b)).

Following 48 h incubation, there was no significant difference in* PR-B* mRNA abundance between vehicle-treated tissues and fresh tissues (Figure 7(c)). Incubating myometrial samples with 2.0, 5.0, or 10.0 *μ*M MG-132 or BAY-11-7085 individually or in combination (10 *μ*M each) had no effect on* PR-B *mRNA abundance relative to vehicle-treated tissues (Figure 7(c)).

Following 48 h incubation, the* PR-A/PR-B* expression ratio was significantly elevated in vehicle-treated tissues compared to fresh tissues (*p* = 0.0011) (Figure 7(d)). Supplementing culture media with 2.0, 5.0, or 10.0 *μ*M MG-132 or BAY-11-7085 individually or in combination (10 *μ*M each) had no effect on* PR-A/PR-B *expression ratio compared to the vehicle-treated tissues (Figure 7(d)).

### 3.8. Controlling Changes in PR Isoform Expression Using TSA

Relative abundance of* PR-T, PR-A, *and* PR-B* mRNA was measured in myometrial tissue incubated for 48 h in the presence of TSA (0.5, 1.0, 2.5, and 5.0 *μ*M) or vehicle (DMSO).

**Figure 8:**
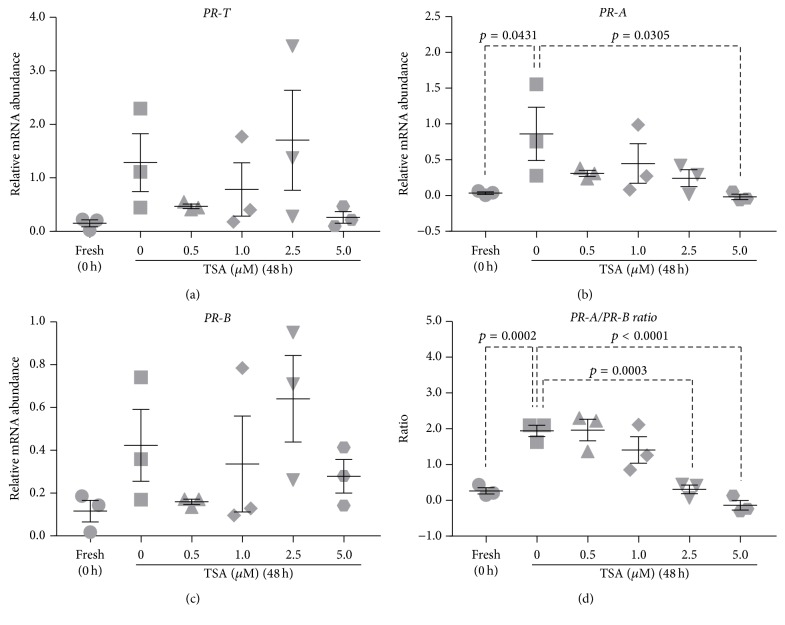
Effect of TSA on culture-induced changes in PR isoform expression in vitro: relative mRNA abundance of* PR-T*,* PR-A,* and* PR-B* was measured in term nonlaboring myometrial tissue samples (*n* = 3) following 48 h incubation in the presence of TSA (0.5, 1.0, 2.5, and 5.0 *μ*M) and expressed relative to Alien reference. In addition,* PR-A/PR-B* expression ratio was calculated. (a)* PR-T* mRNA abundance. (b)* PR-A* mRNA abundance. (c)* PR-B* mRNA abundance. (d)* PR-A/PR-B* expression ratio.* Data was checked for normality (Shapiro-Wilk normality test) and then analysed using 1-way ANOVA with multiple comparisons (Dunnett). Data are mean ± SEM*.

There was no significant difference in* PR-T* mRNA abundance between 48 h vehicle-treated tissues and fresh tissues (Figure 8(a)). Incubating myometrial samples with 0.5, 1.0, 2.5, and 5.0 *μ*M TSA had no effect* PR-T *mRNA abundance relative to 48 h vehicle-treated tissues (Figure 8(a)).

*PR-A* mRNA abundance was significantly increased in 48 h vehicle-treated tissues relative to fresh tissues (*p* = 0.0431) (Figure 8(b)). Culture-induced increases in* PR-A* mRNA abundance were inhibited by supplementing media with TSA. The extent of inhibition reached statistical significance, relative to 48 h vehicle-treated tissue, at 5.0 *μ*M TSA (*p* = 0.0305) (Figure 8(b)).

*PR-B *mRNA abundance did not change following 48 h incubation and was unaffected by TSA treatments relative to vehicle-treated tissues (Figure 8(c)).

Following 48 h incubation, the* PR-A/PR-B* expression ratio was significantly elevated in vehicle-treated tissues compared to fresh tissues (*p* = 0.0002) (Figure 8(d)). The* PR-A/PR-B* expression ratio was significantly lower in tissue treated with 2.5 and 5.0 *μ*M TSA (*p* = 0.0003 and *p* < 0.0001, resp.) relative to 48 h vehicle-treated tissues (Figure 8(d)). TSA dose-dependently prevented culture-induced increases in the* PR-A/PR-B *expression ratio.

## 4. Discussion

Human tissue and cell cultures are a valuable in vitro tool used to investigate the maintenance of uterine quiescence and the mechanisms by which the myometrium transforms to an actively contracting organ at labor. Our previous results show that, upon culturing nonlaboring myometrial tissues in vitro, the tissue undergoes culture-induced changes in expression of the key myometrial genes* ESR1*,* PTGS2,* and* OXTR, *which are consistent with transition toward a procontractile, laboring phenotype (under review). In light of this evidence, we further examined whether* PR* isoform expression undergoes culture-induced changes that are consistent with transition to a procontractile, laboring phenotype.

In this study we examined changes in* PR* isoform expression via determining mRNA levels, an approach which is consistent with other studies in the field [40]. Nevertheless, we are aware that PR isoform protein levels may reflect PR function more closely than mRNA abundance especially in pregnancies complicated by intrauterine inflammation [41]. Previous studies have demonstrated, however, that there is close correspondence between PR isoform mRNA and protein expression changes in the human myometrium at normal term labor [23, 24], which is the context of this study. Furthermore, recent studies examining protein profiles in mammalian cells have found that transcription, not translation, mostly determines protein abundance [42] and that during periods of dynamic change, such as phenotype transition, changes in mRNA abundance play a dominant role in determining changes in protein levels [43]. Overall, assessing myometrial PR function by determining* PR-A* and* PR-B* mRNA levels appears a reliable approach in the patient population we examined.

Tissue incubation studies are routinely performed for 48 h or more [38, 39, 44]. Considering that nonlaboring human myometrium develops contractility in vitro in just 1-2 h [30, 33, 35, 45], the transition away from the original phenotype may be even more pronounced after such 48 h incubations. Our results illustrate that, in human myometrial pieces,* PR-A* mRNA abundance begins increasing after just 1 h culture. With* PR-B* mRNA abundance remaining constant, a statistically significant increase in the* PR-A/PR-B* expression ratio was evident after just 6 h culture (Figure 1). Previous studies using enzyme-immunoassays found that samples collected from the upper segment myometrium during labor had higher total PR concentrations than samples collected prior to labor [46]. Haluska et al. [22] used rhesus monkey, another genus that lacks a systemic progesterone withdrawal, to look at the changes in PR isoform concentrations. They found that there was no change in total PR expression during the transition from late pregnancy to labor; however, they did find a significant shift in the ratio of PR isoforms [22]. More specifically, the myometrial* PR-A/PR-B* ratio increased significantly from late pregnancy to spontaneous labor at term [22]. Furthermore, Pieber et al. [47] performed immunoblot analyses on lower segment myometrium from pregnant women and reported an increase in the PR-A protein abundance during labor, while levels of PR-B were not altered by labor status. Recently, our group showed that the onset of labor is associated with increased abundance of* PR-A* mRNA and an increase in the* PR-A/PR-B* expression ratio in term human myometrium [24]. Our group has also found that the PR-A/PR-B protein ratio in pregnant human myometrium was 0.5 (a PR-B dominant state) at 30 weeks' gestation, which then increased to 1.0 at term prior to the onset of labor, and at the time of the labor the ratio increased further to 3.0 (a PR-A dominant state) [23]. These results indicate that* PR* mRNA levels reflect PR protein levels in human myometrium. Our observation that* PR-T* and* PR-A *mRNA abundance as well as the* PR-A/PR-B* expression ratio increased during culture is therefore consistent with the tissue transitioning to a labor-like state as a consequence of in vitro conditions.

This finding has implications for the interpretation in vitro of studies performed on nonlaboring myometrium, which may have in fact already transitioned to a labor-like phenotype during the early stages of the study and may therefore have affected the outcome of the study. To address this, we sought to identify culture conditions that could be implemented to maintain a nonlaboring state whereby human myometrium retained a low* PR-A/PR-B* expression ratio (a* PR-B* dominant state), thereby providing a more appropriate in vitro model for conducting studies into myometrial biology.

Previous studies utilizing myometrial culture (explants) often included CSS in their media [37–39]. Therefore, we examined whether supplementing culture media with 5% CSS affected culture-induced changes in* PR* isoform expression. Surprisingly, 5% CSS had no significant effect on* PR-T*,* PR-A,* or* PR-B* mRNA abundance after 48 h culture and consequently had no effect on the* PR-A/PR-B *expression ratio (Figure 2). While supplementing media with serum is common practice during in vitro culture, our results indicate that this practice is not sufficient to prevent culture-induced changes in* PR* isoform expression.

The steroid hormone progesterone plays a crucial role in maintaining pregnancy by promoting myometrial quiescence and relaxation [5–7]. In contrast to most mammalian species [8–12], no decrease in maternal serum levels of progesterone can be observed in humans and higher primates prior to the onset of labor [13–15]. Thus, the term “functional progesterone withdrawal” has been used to describe the withdrawal of progesterone action. Once myometrial tissue is removed from in vivo environment and cultured in vitro, the high plasma levels of progesterone are no longer present, which could possibly account for culture-induced changes in* PR* expression in vitro. To explore this, we incubated myometrial tissues in media that contained physiological concentrations of progesterone. Supplementing media with progesterone alone was not sufficient to prevent the culture-induced increases in* PR-T *and* PR-A* mRNA abundance. Moreover, progesterone decreased* PR-B* mRNA abundance; however it was not statistically significant; nevertheless this further exacerbated the increase in the* PR-A/PR-B* expression ratio (Figure 3). A previous study using myometrial strips showed that progesterone exerts rapid inhibition of the amplitude of myometrial contractions in vitro [30]. More recently, Baumbach et al. [31] investigated the suppression of uterine contractility using progesterone alone and in a combination with various tocolytics and found that progesterone alone had little effect inhibiting contractility [31]. This is consistent with our results where progesterone alone did not prevent culture-induced increases in* PR-T *and* PR-A* mRNA abundance.

In numerous mammalian species, the process of parturition, especially transformation of the myometrium from the quiescent to a contractile state, necessitates an increase in circulating estrogen concentrations prior to the onset of labor [11, 14, 48, 49]. In humans and higher primates, however, maternal estrogen levels are high for most of pregnancy and remain elevated during parturition and delivery [14, 50, 51]. Furthermore, our group reported a correlation between estrogen receptor 1* (ESR1)* mRNA levels and the* PR-A/PR-B* mRNA ratio, which is indicative of a functional link between the PR and ESR1 systems [24]. In addition, this link between the two systems is in agreement with studies performed in a range of species demonstrating that progesterone decreases expression of ESR1, thus decreasing uterine responsiveness to estrogen [52, 53]. These results imply that the interaction between progesterone and PR-B suppresses ESR1 expression, therefore rendering the myometrium refractory to circulating estrogen [19]. However, with advancing gestation there is an increase in the expression of PR-A, which in turn represses the transcriptional activity of PR-B, and as a result the PR-B-mediated inhibition of ESR1 expression is withdrawn [19]. Once myometrial tissue is removed from in vivo environment and cultured in vitro, the high plasma levels of progesterone and estrogen are no longer present, thereby removing the functional link between progesterone and estrogen [24] which could possibly account for the observed culture-induced changes in* PR *expression in vitro. To explore this, we incubated myometrial tissue in media that contained physiological concentrations of P4 and E2. The combination of P4 and E2 prevented culture-induced increase in* PR-T* and* PR-A* mRNA abundance observed in vitro. However, P4 in combination with E2 also decreased* PR-B* mRNA abundance; nevertheless this decrease was not statistically significant. As such, after 48 h culture the* PR-A/PR-B* expression ratio had still increased relative to fresh tissue and adopted a* PR-A* dominant state (Figure 3).

Throughout normal pregnancy the uterus increases several-fold in size by both hyperplasia and hypertrophy to accommodate the growing fetus and placenta [54, 55]. A previous study using term nonlaboring human myometrium tissue showed that stretch applied to myometrial cells in culture resulted in decreased* PR-T* and* PR-B* mRNA expression [56]. We found that constant stretch, applied by means of hanging 1 or 3 g weights from tissue strips, had no effect on culture-induced changes in* PR* isoform expression (Figure 4). This is inconsistent with a previous report where stretch downregulated* PR-T *and* PR-B* expression; however, it should be noted that those studies used myometrial cells while our study uses myometrial tissue strips [56]. Previous animal studies suggest that progesterone is responsible for maintaining uterine quiescence and promoting myometrial hyperplasia and hypertrophy to inhibit any increase in uterine wall tension [57–60]. In addition, human studies show that, in a progesterone-dominated endocrine environment, moderate stretch possibly maintains relaxation and quiescence; however, in the absence of progesterone or excessive stretch, the uterus starts to contract [54, 55]. Interestingly, although stretch did not directly affect the culture-induced changes in* PR-T*,* PR-A,* or* PR-B* expression, the application of stretch prevented steroids (P4 + E2) from blocking culture-induced increases in* PR-T *and* PR-A* mRNA expression and prevented steroids (P4 + E2) from decreasing* PR-B* mRNA expression (Figures 3 and 4).

There is now extensive evidence to suggest that components of the cAMP signalling pathway are upregulated in the human myometrium throughout pregnancy to maintain uterine quiescence until term [61–65]. Moreover, our group showed that, in PHM1-31 cells, a pregnant human myometrial cell line, 8-Br-cAMP, an agonist for the protein kinase A (PKA) pathway, increased the expression of both* PR-A* and* PR-B* but had a net effect of decreasing the* PR-A/PR-B* expression ratio [66]. Supplementing media with a cAMP analogue was therefore examined as a potential means to prevent culture-induced changes in* PR *isoform expression. Although cAMP has a well-defined role in promoting myometrial relaxation, supplementing media with 8-Br-cAMP failed to prevent culture-induced changes in* PR-T* or* PR-A *mRNA abundance and increased* PR-A/PR-B* expression ratio (Figure 5).

In contrast to previously discussed treatments that attempted to prevent culture-induced changes in* PR* isoform expression, we also examined the effect of the procontractile agent, PMA, to determine whether* PR* expression would be driven further toward a labor-like state. Previous studies by our group show that PKC activation by PMA increased the* PR-A/PR-B* expression ratio by selectively increasing expression of* PR-A* [66]. This study found that supplementing culture with PMA further increased the* PR-A/PR-B* expression ratio in vitro, which was consistent with this procontractile agent driving further transition toward a laboring phenotype. Interestingly, PMA did not increase expression of* PR-A* but rather decreased expression of* PR-B* over the course of the myometrial culture (Figure 5).

There is increasing evidence that locally produced immune/inflammatory cytokines, particularly prostaglandins (PGs), are involved in normal term labor as well as infection-associated preterm labor [67–69]. In human pregnancy, administration of PGs or PG analogues at any stage of pregnancy transforms the myometrium and cervix and induces labor [69–73]. Previously, our group has tested the hypothesis that PGs, specifically PGF_2*α*_, induce functional progesterone withdrawal by altering myometrial* PR* expression in PHM1-31 cells [66]. PGF_2*α*_ produced a dose-dependent increase in expression of* PR-A*, but not* PR-B*, thereby resulting in an increase in the* PR-A/PR-B* expression ratio [66]. In this study, supplementing media with PGF_2*α*_ had no effect on* PR *mRNA abundance and therefore did not prevent culture-induced changes in the* PR-A/PR-B* expression ratio (Figure 6). This is not consistent with previous results where PGF_2*α*_ increased the* PR-A/PR-B* expression ratio by increasing PR-A expression [66].

Romero et al. [74] have shown that tissue-level inflammation in the myometrium, decidua, and fetal membranes plays a crucial role in the human parturition. In recent years, studies have demonstrated that myometrium in pregnant women at term exhibits biochemical and histological characteristics of inflammation, including increased expression of PGs, increased NF-*κ*B activity, increased infiltration of neutrophils, and macrophages, which may precede the onset of active labor and is independent of infection [6, 8, 75–80]. Furthermore, studies using human myometrial cells have shown that progesterone inhibits the proinflammatory NF-*κ*B transcription factor complex as a result of PR-induced expression of inhibitor-*κ*B-*α* (NFKB1A), a major NF-*κ*B repressor [81]. Supplementing media with NF-*κ*B inhibitors therefore represented a potential means of preventing spontaneous changes in* PR* isoform expression. Supplementing media with MG-132 or BAY-11-7085 had no effect on* PR-T, PR-A, *and* PR-B *mRNA abundance and therefore did not prevent culture-induced changes in the* PR-A/PR-B* expression ratio (Figure 7).

Condon et al. [82] administered TSA, a specific and potent HDACi, to pregnant mice late in gestation and found increased histone H3 acetylation as well as a delay in the initiation of parturition by 24–48 h. Decreased histone acetylation in the pregnant uterus near term, caused by a marked decrease in expression of uterine coactivators with intrinsic histone acetyltransferase activity, might serve an important role in the loss of PR function, thus instigating a functional progesterone withdrawal and the initiation of labor [82]. Furthermore, Wilson et al. [83] used the mouse mammary tumor virus promoter to examine the impact of TSA on PR activated transcription and found that TSA removed the transcription factor nuclear factor 1 from the promoter and decreased PR-induced transcription [83]. Based on these results we hypothesised that TSA may modulate* PR* isoform expression and supplemented culture media with TSA in anticipation of maintaining a low* PR-A/PR-B* expression ratio in vitro. Excitingly, TSA produced a dose-dependent inhibition of culture-induced upregulation of* PR-A* mRNA abundance. With no effect of* PR-B* mRNA abundance, TSA was successful in maintaining a low* PR-A/PR-B* expression ratio over 48 h culture, consistent with freshly isolated term nonlaboring myometrium and consistent with preventing in vitro transformation to a laboring phenotype (Figure 8). Using TSA to maintain a low* PR-A/PR-B* ratio could have important clinical ramifications in that progesterone therapy is currently a leading strategy for the prevention of preterm birth (reviewed by van Zijl et al. [84]). Efficacy of progesterone administration may be enhanced if an agent such as TSA could be administered to preserve or even restore progesterone sensitivity in women with threatened preterm labor.

## 5. Conclusion

Concerns have previously been raised about the ability of primary cells in culture to remain representative of their tissues of origin. Adding to this concern, our previous study shows that term nonlaboring human myometrial tissue undergoes culture-induced changes in expression of* ESR1*,* PTGS2,* and* OXTR *that are consistent with transitioning toward a laboring phenotype. In this study we examined* PR *isoform expression and found that* PR-T* and* PR-A *mRNA expression increased in untreated tissue over 48 h culture. Additionally, the* PR-A/PR-B* expression ratio significantly increased, consistent with transition to a laboring phenotype. Through examining various culture conditions, we were able to maintain a nonlaboring state of PR isoform expression by supplementing culture media with TSA, which prevented the culture-induced increase in* PR-A* mRNA abundance and maintained a low* PR-A/PR-B* expression ratio. In summary, this study demonstrates that

- human myometrial tissues undergo culture-induced upregulation of* PR-T* and* PR-A *mRNA expression, which significantly increases the* PR-A/PR-B* expression ratio in vitro, even in nontreated tissue;
- the combination of progesterone and estrogen downregulated* PR-T* and* PR-A* mRNA expression;
- stretch had no direct effect on* PR-T*,* PR-A, *or* PR-B* expression, but it blocked the effects of progesterone and estrogen on* PR-T and PR-A* expression;
- cAMP was unable to control culture-induced changes in* PR *expression;
- PMA further upregulated* PR-A/PR-B* expression ratio;
- PGF_2*α*_ had no effect of* PR* expression in vitro;
- NF-*κ*B inhibitors were unable to control culture-induced changes in* PR *expression;
- TSA downregulated* PR-A *mRNA expression and downregulated* PR-A/PR-B* expression ratio.

## Figures and Tables

**Table 1:** cDNA primer sequences for *PR-T* and *PR-B*.

Primer	Primer sequence (5′-3′)	Amplicon size	GeneBank #
*PR-T*	F: GTGGGAGCTGTAAGGTCTTCTTTAA R: AACGATGCAGTCATTTCTTCCA	83	NM000926.4
*PR-B*	F: TCGGACACCTTGCCTGAAGT R: CAGGGCCGAGGGAAGAGTAG	68	NM000926.4

*PR-T*, progesterone receptor total; *PR-B*, progesterone receptor isoform B.
